# Circulating Epithelial Cells in Patients with Intraductal Papillary Mucinous Neoplasm of the Pancreas

**DOI:** 10.3390/life13071570

**Published:** 2023-07-15

**Authors:** Jasmina Kuvendjiska, Felix Müller, Peter Bronsert, Sylvia Timme-Bronsert, Stefan Fichtner-Feigl, Birte Kulemann

**Affiliations:** 1 Faculty of Medicine, Albert-Ludwigs-University of Freiburg, 79110 Freiburg im Breisgau, Germany; 2 Department of General and Visceral Surgery, University Medical Center Freiburg, 79106 Freiburg im Breisgau, Germany; 3 Tumorbank, Comprehensive Cancer Center Freiburg, University Medical Center Freiburg, 79106 Freiburg im Breisgau, Germany; 4 Institute for Surgical Pathology, University Medical Center Freiburg, 79106 Freiburg im Breisgau, Germany; 5 Department of Surgery, University Medical Center Schleswig-Holstein, 23538 Lübeck, Germany

**Keywords:** circulating epithelial cells, circulating tumor cells, intraductal papillary mucinous neoplasm, IPMN, KRAS, liquid biopsy, liquid biomarker

## Abstract

Intraductal papillary mucinous neoplasm (IPMN) is the most common pancreatic cyst and a precursor of pancreatic cancer (PDAC). Since PDAC has a devastatingly high mortality rate, the early diagnosis and treatment of any precursor lesion are rational. The safety of the existing guidelines on the clinical management of IPMN has been criticized due to unsatisfactory sensitivity and specificity, showing the need for further markers. Blood obtained from patients with IPMN was therefore subjected to size-based isolation of circulating epithelial cells (CECs). We isolated CECs and evaluated their cytological characteristics. Additionally, we compared Kirsten rat sarcoma viral oncogene homolog (KRAS) mutations in CECs and the primary IPMN tissue, since KRAS mutations are very typical for PDAC. Samples from 27 IPMN patients were analyzed. In 10 (37%) patients, CECs were isolated and showed a hybrid pattern of surface markers involving both epithelial and mesenchymal markers, suggesting a possible EMT process of the cells. Especially, patients with high-grade dysplasia in the main specimen were all CEC-positive. KRAS mutations were also present in CECs but less common than in IPMN tissue. The existence of CEC in IPMN patients offers additional blood-based research possibilities for IMPN biology.

## 1. Introduction

The incidence of pancreatic cystic lesions is increasing partially due to better detection by imaging techniques [1]. The most common type of pancreatic cyst is the intraductal papillary mucinous neoplasm (IPMN), a mucin-producing pancreatic cyst arising from the pancreatic duct system [2,3]. There are three main types of IPMN, based on the association of the cyst with the pancreatic duct: main-duct IPMN (MD-IPMN), branch-duct IPMN (BD-IPMN), and mixed-type IPMN (MT-IPMN) [4]. Histologically, IPMN can be categorized into gastric, intestinal, and pancreatobiliary types [5]. Oncocytic IPMN is now classified as a separate entity by the 2019 WHO classification [6]. All types of IPMN are considered possible precursors of pancreatic cancer, with an especially high malignancy potential in MD-IPMN [7]. Since pancreatic cancer still has a devastating prognosis with a 5-year survival rate of 3–15% [8,9], the early diagnosis and treatment of any potential precursor lesions are of great importance. Different morphological characteristics as well as clinical symptoms are currently used for stratification and decision making regarding the therapy of IPMN. It is generally recommended that MD-IPMN be resected due to the high malignancy risk. On the other hand, there remain some controversy and insecurity regarding the treatment of BD-IPMN. Also, it is sometimes challenging to evaluate the main duct involvement before surgery. Different consensus criteria, like the Fukuoka Consensus Guidelines (FCG) [10] and the Sendai Consensus Guidelines (SCG) [11], have been established to help clinicians decide whether resection is indicated or surveillance is sufficient (Table 1). Ideally, unnecessary risky operations should be avoided in benign cases, as should long surveillance in (undetected) high-risk IPMN with a greater risk of malignant transformation into pancreatic cancer. The safety of these criteria has been questioned by different studies [12,13]. A systematic review by Srinivasan et al. showed a higher positive predictive value of the FCG (42%) compared to the SCG (33%) and a slightly lower negative predictive value of the FCG (86%) compared to the SCG (90%) [12]. Thus, malignant IPMN may be missed according to both guidelines, and patients with benign lesions might undergo unnecessary surgery. This underlines the urgent necessity for better pre-therapeutic stratification methods. 

The transition from IPMN to cancer is not fully understood, but it progresses from low-grade dysplasia followed by high-grade dysplasia and ends in invasive cancer. Generally, there are two theoretical models of systemic cancer progression: linear and parallel [14]. The linear model places the driver of cancer progression within the primary tumor before metastatic dissemination of fully malignant cells. The second model posits parallel, independent progression of metastases arising from early-disseminated tumor cells [14]. According to the parallel model of progression, cells can detach from the original tissue and enter the bloodstream even before malignancy can be detected [15]. One of the proposed mechanisms is the so-called epithelial-to-mesenchymal transition (EMT), in which cells lose the epithelial markers and develop mesenchymal or stem cell markers. Using a mouse model of pancreatic cancer, Rhim et al. showed cells that entered the bloodstream even before any malignancy could be detected by histologic analysis [15]. These circulating pancreatic cells were associated with EMT and maintained a mesenchymal phenotype, exhibited stem cell properties, and seeded the liver. The same group confirmed these findings by detecting circulating pancreas epithelial cells in blood samples of patients with cystic lesions and no clinical diagnosis of cancer [16]. Today, the circulating epithelial or cancer cells are widely examined as part of liquid biopsy in different cancer types but also in non-malignant precursor lesions. Several studies have described the existence of circulating tumor cells (CTCs) in patients with pancreatic carcinoma and put it in connection with worse progression-free survival (PFS) and overall survival (OS) [17,18,19,20,21]. The available data about the existence and clinical relevance of CEC in patients with non-malignant pancreatic lesions are still very limited [22,23]. In 2017, Poruk et al. published one of the first studies showing the possible clinical relevance of CEC in patients with IPMN in which CEC-positive patients had significantly more high-grade dysplasia [23].

On the genetic level, some of the described pathways for the malignant transformation of IPMN are KRAS, GNAS, TP53, and SMAD4 mutations. KRAS is of special interest in pancreatic carcinoma since more than 90% of pancreatic carcinomas and even non-invasive, low-grade dysplasia PanINs show a KRAS mutation [24]. KRAS mutations could be proven not only in the primary tumor/lesion but also in CTCs in patients with pancreatic carcinoma [19], often with different mutations than the originating tumor. Data about possible KRAS mutations in CEC of IPMN patients are still lacking, while a high prevalence of KRAS mutations in IPMN tissue has already been demonstrated. 

With reference to the above, the following questions arise: Are there factors that promote the occurrence of CEC in patients with IPMN? Which markers are expressed on the surface of CECs, and is there any expression of mesenchymal (EMT-triggered) markers? Do CECs in IPMN patients show any KRAS mutations?

The goal of this study was to determine the existence of CECs in patients with IPMN and describe their surface marker expression as well as their possible KRAS mutations.

## 2. Materials and Methods

### 2.1. Patient Selection

This study was performed as a monocentric study in the Department of General and Visceral Surgery, University Medical Centre Freiburg, Germany, and was approved by the Ethics Committee of the Albert-Ludwigs-University Freiburg (371/14) Freiburg, Germany. All patients gave full informed consent for materials, data acquisition, and experiments. 

We enrolled all patients with suspected IPMN scheduled for pancreatic resection from February 2014 until November 2019. Clinical follow-up was carried out until 2 years after the last patient was included. Patient data including medical history, disease symptoms, and laboratory results were extracted from the patient charts.

The inclusion criteria were (a) clinically suspected IPMN, (b) patient scheduled for any type of pancreatic resection, (c) age > 18 years, and (d) no history of malignant tumor in the past 5 years. Patients with histological dismissal of the initially presumed diagnosis of IPMN were excluded from the study. Moreover, patients with histological proof of a malignant pancreatic tumor in the surgical specimen were excluded from further investigation. The inclusion criteria for the control patients were (a) age > 18 years, (b) no history of malignant tumor, and (c) no known pathology of the pancreas (malignant or non-malignant). 

Blood specimens were sampled before surgery (two EDTA tubes). The first 20 mL of blood was used for routine purposes in order to minimize the risk of contamination of the specimen with epithelial skin cells during puncture. Prompt cell and DNA isolation were undertaken following the blood collection by using the ScreenCell^®^ isolation system. ScreenCell^®^ offers different isolation kits depending on the planned downstream analysis. The ScreenCell^®^ Cyto devices are designed for the isolation of fixed cells for cytological studies and the ScreenCell^®^ MB devices are designed for the isolation of live cells for molecular biology. Circular pores are calibrated at 7.5 ± 0.36 μm for the isolation of fixed cells and at 6.5 ± 0.33 μm for live cells. Since we performed a phenotypical characterization of the cells by cytomorphology and immunocytology, as well as molecular characterization of the CECs, we used two different kits for CEC isolation: ScreenCell^®^ Cyto and ScreenCell^®^ MB. Further analyses were performed after the IMPN was histologically proven in the operatively removed pancreatic specimen. 

### 2.2. CEC Isolation Method and Cytological Evaluation

For cytological characterization of CEC, 6 mL of EDTA blood was processed within 4 h of draw (3 mL blood per 1 filtration device) through two ScreenCell^®^ Cyto Kit filtration devices (ScreenCell SA, Sarcelles, France) according to the manufacturer’s instructions, as previously described [25,26]. In brief, to fix the cells and lyse red blood cells, 3 mL of blood was diluted in 4 mL of filtration buffer ScreenCell-fixed cells (FC2) and incubated for 8 min before filtration. Subsequently, the diluted blood was passed through the ScreenCell^®^ filter. The ScreenCell^®^ system is fitted with microfilters (filter pore size 7.5 µm) that capture the cells on small metal-rimmed filters via low-pressure vacuum filtration. This represents a surface-marker-independent CEC enrichment method, allowing the isolation of unmodified cells for downstream analysis, and it has been used in liquid biopsy research in different cancer types [19,25,27,28,29].

The isolated CECs were fixed, permeabilized, and stained with Hoechst 33342 (Thermo Fisher Scientific Inc., Waltham, MA, USA) and an antibody against EpCAM (ab232539), L1CAM (ab24345), vimentin (M0725, Dako Denmark, Glostrup, Denmark), and PDX1 (ab240084, Abcam plc., Cambridge, UK). EpCAM represents an epithelial marker expressed only in epithelia and epithelial-derived neoplasms. Vimentin is expressed in mesenchymal cells and is often used as a marker of mesenchymal-derived cells or cells undergoing an epithelial-to-mesenchymal transition (EMT). Similarly, L1CAM is relevant for the progression of tumors and has been put in connection with EMT processes. PDX1 is a pancreas-specific transcription factor. Every filter (two filters per patient) was dual-stained with one of the following combinations: anti-EpCAM/-L1CAM or anti-PDX1/-vimentin, as described below.

Step 1: Filters were dried for 1 h at 37 °C. Step 2: Isolated cells were permeabilized on the filters using 0.5% Triton X-100 (TRX, Sigma T8787) for 5 min in the case of EpCAM/-L1CAM dual staining or 20 min for anti-PDX1/-vimentin dual staining. Step 3: Filters were washed three times with Dulbecco’s Phosphate-Buffered Saline (DPBS) and then blocked with 2% goat serum (diluted in DPBS) for 30 min. Step 4: Incubation at 4 °C overnight with primary antibody diluted in 2% goat serum (anti-EpCAM antibody 1:100 and anti-L1CAM antibody 1:500) or for 30 min at room temperature with primary antibody diluted in 2% goat serum (anti-PDX1 antibody 1:100 and anti-vimentin antibody 1:500). Step 5: Filters were then washed four times with DPBS and subsequently incubated for one hour at room temperature with fluorescent-labeled secondary antibodies: Cyanine3 (Goat anti-Rabbit IgG Cyanine3, A10520, Thermo Fisher, Waltham, MA, USA)/Alexa Fluor^®^ 488 (Goat anti-Mouse IgG Alexa Fluor^®^ 488 A11029, Thermo Fisher, Waltham, MA, USA), 1:500 diluted in 2% goat serum (Sigma-Aldrich, Burlington, MA, USA). Step 6: Next, filters were washed three times with DPBS followed by nuclear staining with Hoechst 33342 (1:10,000 diluted in distilled water) for 3 min. Step 7: Lastly, filters were washed two times with distilled water and dried at room temperature until microscopic evaluation.

Positive and negative controls for the immunofluorescence stains were realized using in vitro cultivated cell lines as follows: pancreatic adenocarcinoma cell line HPAF-II (ATCC, Manassas, VA, USA) as a positive control for EpCAM, L1CAM, and PDX1 expression; melanoma cell line MelIm as a positive control for vimentin expression. The latter cell line was also used as a negative control for EpCAM and PDX1 expression. The cell line H6C7 (Kerafast, Boston, MA, USA) was used as a negative control for L1CAM expression and HPAF-II for vimentin expression.

Suspected CECs were then identified and photographed under a fluorescence microscope (Olympus BX61, Olympus DP80). The staining intensity was graded as negative, low, moderate, and strong.

Subsequently, cell cytology was visualized with either Giemsa staining (Merck KGaA, Darmstadt, Germany) according to the ScreenCell^®^ protocol (protocol PR_A02 MGG, version: 14.08.2015) or HE stain using Hemacolor^®^-Kit (Merck KGaA) and independently re-evaluated via a bright-field microscope by two pathologists. CECs were defined according to the cytological criteria by Rosenbaum et al. [20]: cells over 2 times the pore size, with either irregular, hyperchromatic nuclei and scant cytoplasm or clusters of cells with round/oval nuclei with occasional grooves and visible cytoplasm; suspicious CECs were epithelioid cells but fell short of the Rosenbaum criteria or lacked clear cytoplasm (naked nuclei). No cut-off was chosen for CEC evaluation to maintain a complete picture. 

### 2.3. CEC and Tissue DNA Isolation and KRAS Genotyping

For genetic analysis of CECs, 6 mL of EDTA blood was filtered using an additional ScreenCell^®^ MB Kit (ScreenCell SA, Sarcelles, France) (filter pore size 6.5 µm) according to the manufacturer’s instructions. Using the QIAamp^®^ DNA Micro Kits (QIAGEN, Hilden, Germany), the DNA from the captured cells was isolated from the filter according to the manufacturer’s instructions.

For the IPMN tissue DNA, three 10 µm thick FFPE sections from the operatively removed specimen were used. Areas containing IPMN were microscopically identified and manually macro-dissected. Lastly, the tissue DNA from the macro-dissected samples was extracted via the QIAamp^®^ DNA FFPE Tissue Kit (QIAGEN, Hilden, Germany).

Multitarget Droplet Digital PCR was used to validate selected mutations in KRAS (G12D, G12V, G12C, G12R, and G12A), according to Hussung et al. [30] by multitarget and control assays. Fluorescence amplitude was analyzed using the QX100™ Droplet Reader (Bio-Rad Laboratories, Hercules, CA, USA) and QuantaSoft^®^ Software Version 1.7.4.0917 (Bio-Rad Laboratories).

### 2.4. Histological Evaluation of the IPMN Tissue

For comparison with the isolated CECs, the removed IPMN tissue was immunohistologically analyzed for the expression of the markers EpCAM (ab232539), L1CAM (ab24345), PDX1 (ab240084; Abcam plc., Cambridge, UK), and vimentin (IR630, Dako Denmark). For this, 4 μm thick FFPE tissue sections were generated and stained with Mayer’s hemalaun solution (Waldeck GmbH & Co., Münster, Germany) and an antibody against EpCAM, L1CAM, PDX1, and vimentin in the following steps: 

Step 1: Tissue sections were dried at 56 °C overnight. Step 2: The sections were then deparaffinized with Xylol (2 × 25 min), rehydrated with ethanol in decreasing concentrations (2 × 5 min 100%, followed by 90%, 70%, and 50% for 5 min each), and washed twice with distilled water. Step 3: For antigen retrieval, sections were placed into preheated Target Retrieval Solution (Dako S1699, Dako Denmark) and incubated for 30 min in a steamer (850 Watt). Step 4: The sections remained in the Target Retrieval Solution (Dako S1699) for 10 more min while cooling down in ice water and were then washed once with distilled water. Step 5 *: Incubation with EnVision Flex Peroxidase-Blocking Reagent (Dako Denmark) for 10 min. Step 6 *: Incubation with primary antibody (anti-PDX1 antibody: 6 µg/mL (diluted in Zytomed Antibody Diluent) for 1 h; anti-vimentin antibody: RTU for 20 min; anti-EpCAM antibody: 5 µg/mL (diluted in Zytomed Antibody Diluent) for 1 h; anti-L1CAM antibody: 0.25 µg/mL (diluted in Zytomed Antibody Diluent) for 10 min). Step 7 *: Incubation with EnVision FLEX+ Linker for 15 min. Step 8 *: Incubation with EnVision FLEX/HRP for 20 min. Step 9: Staining was visualized using incubation with EnVision FLEX DAB + Chromogen (diluted 1:51 in EnVision FLEX Substrate Buffer) for 10 min. Step 10: Sections were washed with distilled water. Step 11: Counterstaining was carried out with Mayer’s hemalaun solution for 30 s; excess staining solution was removed with distilled water. Step 12: Sections were subsequently dehydrated with 100% ethanol (4 × 3 s) and stabilized with Xylol (2 × 3 s). Step 13: Lastly, sections were covered with foil using Xylol as mounting medium.

* After this step (steps 5, 6, 7, and 8), sections were washed with Dako Wash Buffer (Dako Denmark) for 5 min (in the case of anti-L1CAM staining, Dako PBS was used in place of Dako Wash Buffer).

For quality assurance of the stain results, control immunohistology was performed on non-pancreatic tissue (appendix vermiformis, tuba uterine, duodenum, tonsils, thyroid, and smooth muscle tissue). The tissues were obtained from the Biobank, Comprehensive Cancer Centre, University Medical Centre Freiburg, Germany.

The stained tissue sections were scanned (Pannoramic SCAN^®^ Slide Scanners, Software: Pannoramic Scanner, SlideViewer, Version 2.6) for microscopic evaluation and the immunoreactive score (IRS) was applied to assess the immunostaining extent [31]. The IRS is composed of the staining intensity (0 = negative, 1 = low, 2 = moderate, 3 = strong) multiplied by the percentage of stained cells (0% = 0, ≤10% = 1, 11–50% = 2, 51–80% = 3, ≥81% = 4). The IRS ranges from 0 to 2 (negative), 3 to 4 (low), 6 to 8 (moderate), and 9 to 12 (strong). The evaluation was carried out by two different pathologists. 

### 2.5. Statistics

Descriptive statistics were applied to the patient characteristics. Categorical data were summarized by absolute and relative frequencies. Continuous data were summarized by mean, standard deviation, median, quartiles, and range. Statistical analysis was performed using IBM SPSS Statistics version 28.0 (IBM Corp, Armonk, NY, USA). Categorical data were analyzed in contingency tables using Pearson’s chi-squared test and Fisher’s exact test. For relations between categorical and continuous data, a quantile–quantile plot was first used to determine the normal distribution of the continuous data. Further analysis was conducted with the *t*-test or ANOVA for data with normal distribution and the Mann–Whitney U test or the Kruskal–Wallis test for data without normal distribution. *p*-values of *p* < 0.05 were considered significant.

## 3. Results

### 3.1. Study Population

Overall, 62 patients were included in the study after the initial screening. Following the final histology of the operatively removed specimen, 28 patients had to be excluded from further examination due to the histological dismissal of IPMN. Five patients were excluded due to an underlying secondary malignancy and another two patients due to incomplete data. Lastly, 27 patients were included in the final analysis, of which 48% (n = 13/27) of patients were male and 52% (n = 14/27) were female. Their average age was 66 years. All patients had histologically proven IPMN, of which 25.9% were classified as main-duct IPMN, 51.9% as mixed-type IPMN, and 22.2% as branch-duct IPMN. The mean size of the largest cyst was 16.9 mm (SD: 8.48 mm). Histological characteristics and dysplasia grade are shown in Table 2.

The indication for surgery was given immediately in 51.9% of the cases and in 48.2% after cyst-size increase during clinical follow-up or the occurrence of worrisome features (median follow-up time: 21.3 months; SD: 17.9 months). The tumor marker CA19-9 was measured preoperatively in 81.5% of the patients, of whom only 13.6% showed pathologically increased values > 27 U/mL (mean 19.6 U/mL, SD 32.7 U/mL).

The five patients in the control group (three male, two female, mean age: 41 years) had no history of cancer or any diseases of the pancreas. 

### 3.2. Histological Evaluation of the IPMN Tissue

**Figure 1:**
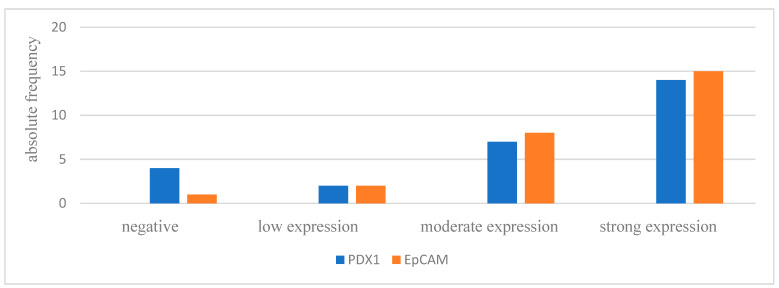
IPMN tissue IHC: intensity of PDX1 and EpCAM expression. IRS = immunoreactive score: negative = IRS 0–2; low expression = IRS 3–4; moderate expression = IRS 6–8; strong expression = IRS 9–12.

**Figure 2:**
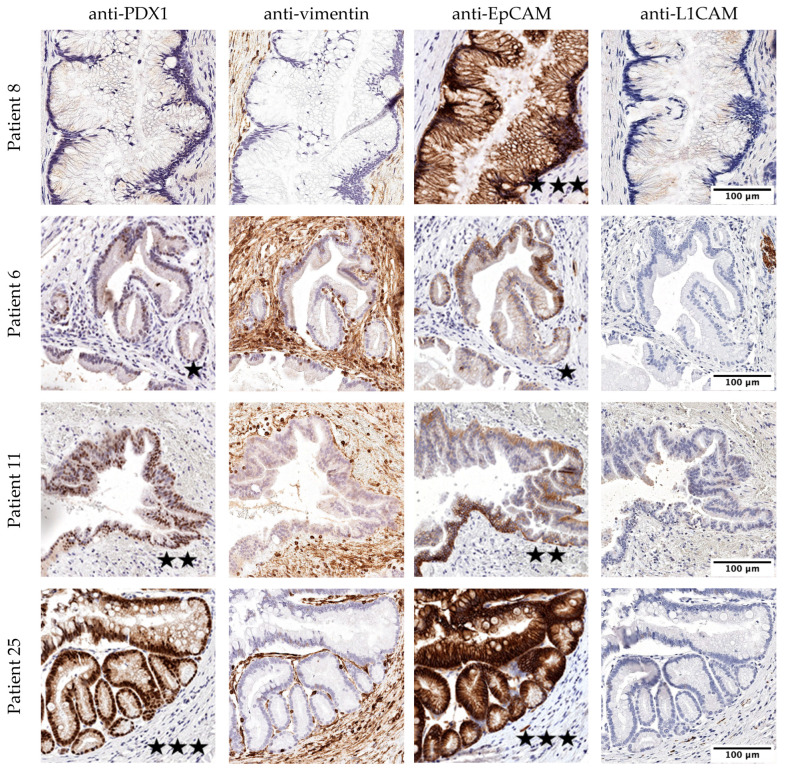
IHC stains on resected IPMN tissue. Exemplary pictures of IHC stains of the resected IMPN tissue. Patient 8: PDX1 negative, vimentin negative, EpCAM strong expression, L1CAM negative. Patient 6: PDX1 low expression, vimentin negative, EpCAM low, L1CAM negative. Patient 11: PDX1 moderate expression, vimentin negative, EpCAM moderate expression, L1CAM negative. Patient 25: PDX1 strong expression, vimentin negative, EpCAM strong, L1CAM negative. Low expression marked with one star; moderate expression marked with two stars; strong expression marked with three stars.

All 27 patients showed negative IHC stains of the mesenchymal markers L1CAM and vimentin in the removed IPMN tissue. On the other hand, all patients except one were positive for EpCAM, and 85.2% of the patients were positive for the pancreas-specific marker PDX1. Expression intensity is shown in Figure 1. Exemplary pictures of the IHC are shown in Figure 2. Positive and negative control immunohistology was performed on non-pancreatic tissue (Supplementary Material). 

### 3.3. CEC Isolation Method and Cytological Evaluation

**Figure 3:**
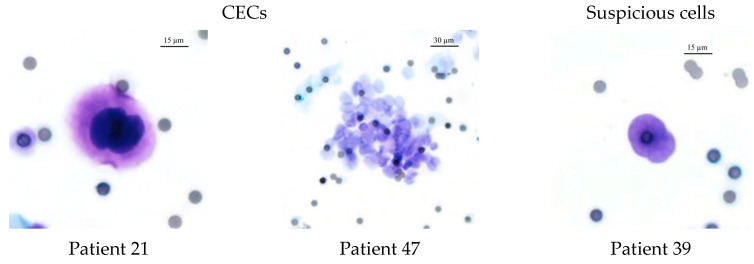
Exemplary pictures of HE stain of CEC, cluster, and suspicious cells. Patient 21: single markedly enlarged cell with nuclear enlargement (>3× pore size), nuclear hyperchromasia, and nuclear membrane irregularity; Patient 47: cluster of epithelioid cells; Patient 39: suspicious cell with markedly enlarged, irregular nuclei but no visible cytoplasm. Cells were isolated by ScreenCell^®^, HE stain (20× magnified). Filter pores (7.5 μm).

CECs were isolated in 37% (n = 10) of the patients and suspicious cells or naked nuclei were identified in 48% (n = 13) of the patients (Figure 3). The remaining 15% (n = 4) of the patients had neither CECs nor any suspicious cells. Five (50%) of the ten CEC-positive patients had clusters in addition to the single CECs. None of the control patients showed cells that fulfilled the CEC criteria.

CEC positivity in relation to histological or genetic characteristics of IPMN is summarized in Table 3. The patients with high-grade dysplasia were CEC-positive. In addition, patients with KRAS mutation in the tissue were often CEC-positive or had suspicious cells. 

**Figure 4:**
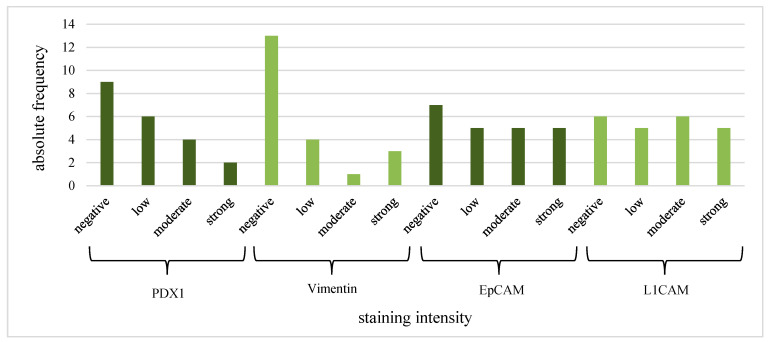
CEC IF: intensity of PDX1, vimentin, EpCAM, and L1CAM expression.

**Figure 5:**
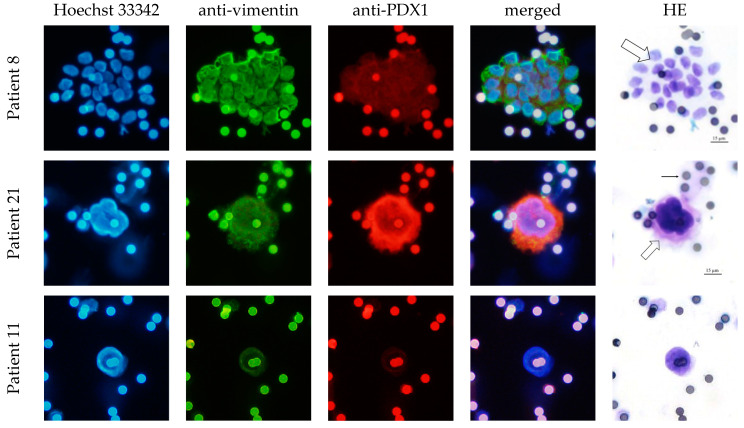
PDX1 and vimentin expression in CECs of IPMN patients. Exemplary pictures of the immunofluorescence labeling of cluster CECs (merged group of cells, marked with block arrow; Patient 8) and single CEC (single very enlarged cell, marked with block arrow; Patient 21 and Patient 11). Cells isolated by ScreenCell^®^; 20× magnified; scale bar 15 µm. Filter pores (7.5 µm) exemplary marked with simple black arrow; the pores of the filters show autofluorescence. HE = hematoxylin–eosin stain. The cluster shows a strong vimentin expression and moderate PDX1 expression. The single CEC in Patient 21 shows moderate vimentin and strong PDX1 expression. CEC in Patient 11 shows moderate vimentin and no PDX1 expression.

**Figure 6:**
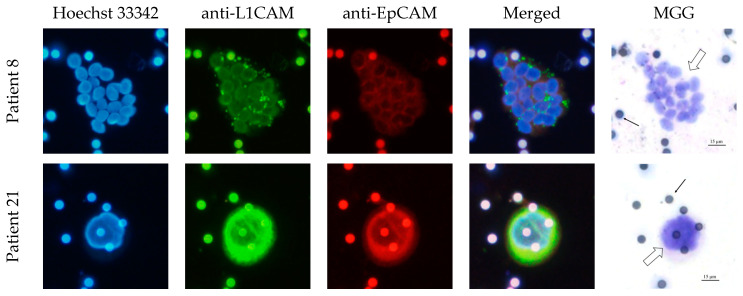
EpCAM and L1CAM expression in CECs of IPMN patients. Exemplary pictures of the immunofluorescence labeling of cluster CECs (merged group of cells, marked with block arrow, Patient 8) and single CEC (single very enlarged cell, marked with block arrow, Patient 21). Cells isolated by ScreenCell^®^; 20× magnified; scale bar 15 µm. Filter pores (7.5 µm) marked with simple black arrow. The pores of the filters show autofluorescence. MGG = May–Grünwald–Giemsa stain. Both the cluster and the CEC presented here show strong L1CAM expression and moderate EpCAM expression.

**Figure 7:**
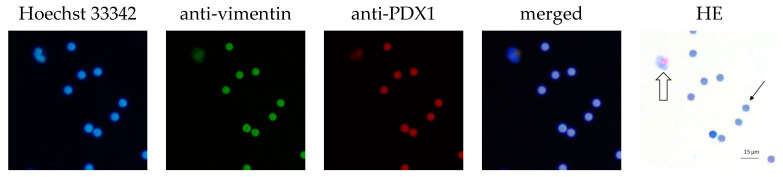
PDX1 and vimentin expression in WBC of IPMN patients. Exemplary pictures of the immunofluorescence labeling of white blood cell (marked with block arrow). Cell isolated by ScreenCell^®^; 20× magnified; scale bar 15 µm. Filter pores (7.5 µm) exemplary marked with simple black arrow; the pores of the filters show autofluorescence. HE = hematoxylin–eosin stain. The white blood cell shows no vimentin expression and no PDX1 expression.

In total, 77.8% of the CECs showed positive expression of PDX1 and vimentin. The clusters were all vimentin- and PDX1-positive. Furthermore, 80% of the CECs and all clusters were EpCAM- and L1CAM-positive (Table 4). The intensity of the IF stain is shown in Figure 4. Exemplary pictures of the IF are shown in Figure 5 and Figure 6. The expression of L1CAM showed a significant correlation to the expression of PDX1 (*p* = 0.008) and EpCAM (*p* < 0.001) but no correlation to vimentin (*p* = 0.055). The expression of EpCAM was significantly correlated with the expression of vimentin (*p* = 0.042) and PDX1 (*p* = 0.049). There was no correlation between the expression of PDX1 and vimentin (*p* = 0.067). Exemplary pictures of the positive and negative control stains on the cell lines HPAF-II, MelIm, and H6C7 are included in the Supplementary Material. White blood cells (WBCs) showed no expression of PDX1, EpCAM, and L1CAM in control stains (Supplementary Material) as well as in rarely residual WBC on the filters (Figure 7). Some WBCs in the control stains showed positive vimentin expression. The vimentin positivity in some WBCs is to be expected since neutrophils and lymphocytes are known to express vimentin.

### 3.4. KRAS Mutation Analysis

**Figure 8:**
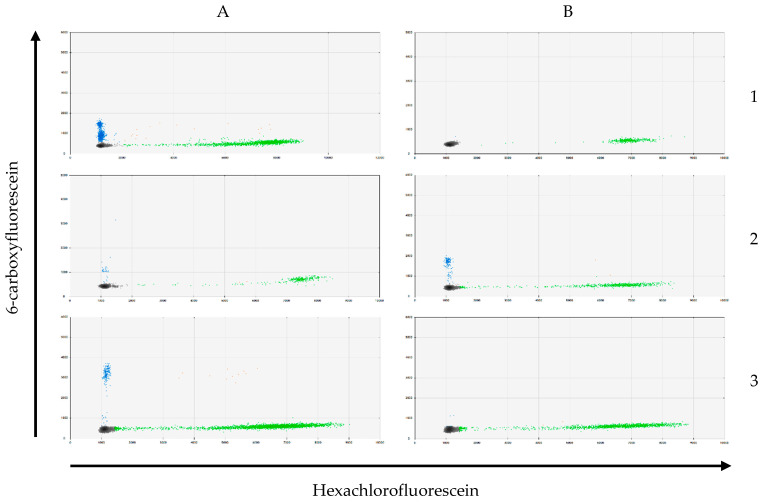
Exemplary picture of KRAS mutation analysis by multitarget Droplet Digital PCR. Gray = droplets without DNA; blue = droplets with mutated DNA; green = droplets with wild-type DNA; orange = droplets with mutated and wild-type DNA; IP = IPMN patients; K = control patients. (**A1**) IP (blood sample DNA) = KRAS genotype G12D/-R/-A; (**B1**) K (blood sample DNA) = KRAS wild-type; (**A2**) IP (tissue DNA) = KRAS genotype G12D/-R/-A; (**B2**) IP (tissue DNA) = KRAS genotype G12V; (**A3**) IP (tissue DNA) = KRAS genotype G12C; (**B3**) IP (tissue DNA) = KRAS wild-type.

KRAS mutation analysis was carried out on DNA from IPMN tissue (n = 27) and on CEC DNA extracted from the blood samples (n = 21). KRAS mutation was proven in 19 tissue samples (70.4%) by multitarget Droplet Digital PCR (Figure 8). The most common mutation was G12D, -R or -A (57.9%), followed by the mutation of G12V (31.6%) and lastly, G12C (10.5%). 

As expected, KRAS mutations in CECs were infrequent among the blood specimens: they were present in only 23.8% (n = 5) of the blood samples (G12C (n = 1), G12V (n = 1), G12D, -R or -A (n = 3)). Blood samples from the control group showed no KRAS mutations. Of the five patients with proven KRAS mutation in the CECs, only three had the same KRAS mutation in the tissue. There was no statistically significant connection between the KRAS mutational status in the blood samples and any histological or morphological characteristics of the IPMN (Table 5).

## 4. Discussion

In the present study, we isolated CECs in patients with IPMN and subsequently evaluated their cytological and genetic characteristics in comparison with the original resected IPMN tissue. Almost half of the patients showed suspicious cells, but CECs were found in only 37% of the included patients. Cluster CECs were found in 50% of the CEC-positive patients. By contrast, no CECs were found in the blood samples of the control patients. 

The presence of CECs in the bloodstream did not show any relevant connection to the radiological or histological characteristics of the IMPN. This is comparable to the findings of Poruk et al., who reported a significantly higher occurrence of CECs in IPMN patients with high-grade dysplasia [23]. Likewise, in our study, all patients with high-grade dysplasia were CEC-positive. 

KRAS mutations and surface marker expression also showed discordant results in CEC and tissue analysis. Particularly, the expression of mesenchymal marker proteins such as vimentin and L1CAM were frequently found in CECs but never in the originating IPMN tissue. The tissue showed mainly an expression of the epithelial surface marker EpCAM and the pancreas-specific marker PDX1, suggesting a high degree of differentiation. Lahat et al. demonstrated a correlation between the increased expression of mesenchymal markers in IPMN tissue and higher-grade dysplasia as a sign of EMT [32]. The IHC results in our study might be affected by the small portion of patients with high-grade dysplasia in the study collective. On the other hand, the CECs showed either no expression of the investigated surface markers or a hybrid (mesenchymal and epithelial) expression of the above-mentioned markers. Furthermore, all cluster CECs were positive for all investigated surface markers. The expression patterns of surface markers involving only epithelial markers in the IPMN tissue and both mesenchymal and epithelial markers in CEC and clusters suggest a potential EMT of the cells.

Regarding the KRAS mutational status, we observed the presence of KRAS mutation in the CECs, although with a much lower incidence than in the IPMN tissue. Nevertheless, this is the first study that reports on the presence of KRAS mutations in CECs in patients with IPMN. Notably, the presence of CECs was much higher in patients with KRAS mutations than in patients with wild-type KRAS. Since KRAS mutations are present in over 90% of PDAC and considered to be an early event in the development of PDAC [33,34], this could be considered a sign of a higher occurrence of CECs in patients with premalignant IPMN lesions. Due to the small collective size and relatively rare occurrence of KRAS mutations in the CECs, the clinical impact of this finding cannot be determined in this study and requires further examination. The mere existence of KRAS mutations in CECs is of great interest since it points out that at least some of the CECs have the potential to develop “malignant behavior”. We already reported on the presence of heterogeneous KRAS mutations in CTCs in patients with PDAC with often discordant mutations from the originating tumor [19]. Some of them were surprisingly even associated with better OS compared to other KRAS mutations [19]. Not only the CTCs but also the primary tumors are known to often harbor more than one single mutation. The heterogeneity of mutations in CTC and primary lesion PDAC—and now also IMPN and CEC—demands further examination regarding the clinical impact of the mutational status. Especially, in the reported CECs in patients with IPMN, this could provide additional information about the malignant tendency of IPMN and help clinicians in treatment decision making.

The present study has several limitations. First, the limited sample size does not allow any meaningful correlations with patient survival rates or other clinical impacts of the CECs. For this, further larger longitudinal studies are required. Second, there were very few patients with high-grade dysplasia in the study, so this important subset of patients is underrepresented in this study, possibly leading to false lower numbers of CECs. This might be of impact especially in the KRAS blood analysis, since we had no blood samples for the analysis of patients with high-grade dysplasia. At the same time, all patients with high-grade dysplasia in their tumor specimens also showed CEC in their blood. Despite the evidence of cytomorphology and IF staining, there remains a residual uncertainty about the origin of the cells. Due to a known vimentin expression in some WBCs, a potential false interpretation cannot be excluded if a CEC-suspected cell is only vimentin-positive. Since the cells’ categorization was based not only on the immunofluorescence labeling but also on the morphological features visible in the HE or MGG stain, such misinterpretation risk should be minimized.

To our knowledge, this is the largest study on CECs in IPMN. Furthermore, this is the first study describing the presence of KRAS mutations in CECs, which might be a sign of the malignant potential of IPMN. Further studies are needed to define the clinical impact of CECs and KRAS mutations in CECs as well as their possible clinical implications in risk stratification and therapy of IPMN.

## 5. Conclusions

CECs are present in the blood of IPMN patients. In this study, they were found in all patients with high-grade dysplasia in the main specimen. The surface marker expression of CECs often shows a hybrid pattern involving epithelial and mesenchymal markers and is discordant with the expression pattern of the originating IPMN tissue. This might be due to EMT processes. KRAS mutations, typical for PDAC, are detectable in CECs but less common than in primary IPMN tissue. Further studies are needed to evaluate the clinical utilization of CEC in patients with IPMN. 

## Figures and Tables

**Table 1:** Resection criteria according to the Sendai and Fukuoka Consensus Guidelines; MPD = main pancreatic duct.

Therapy Recommendation	Sendai Consensus	Fukuoka Consensus	
Resection	cyst size > 3 cm presence of mural nodules positive cytology clinical symptoms MPD dilation	High-risk stigmata	obstructive jaundice enhancing mural nodule ≥ 5 mm MPD diameter ≥ 10 mm
Surveillance		Worrisome features	MPD diameter 5–9 mm cyst diameter ≥ 30 mm enhancing mural nodules < 5 mm thickened/enhancing cyst walls IPMN-induced acute pancreatitis increased serum level of CA 19-9 cyst growth rate ≥ 5 mm/2 years lymphadenopathy MPD stenosis with distal atrophy

**Table 2:** Histological characteristics of IPMN and dysplasia grade.

		Maximal Dysplasia Grade in IPMN			
		Low-Grade	Intermediate-Grade	High-Grade	Total
		n (%)	n (%)	n (%)	n (%)
IPMN type	branch-duct	4 (66.7%)	2 (33.3%)	0 (0%)	6 (22.2%)
	mixed-type	10 (71.4%)	2 (14.3%)	2 (14.3%)	14 (51.8%)
	main-duct	6 (85.7%)	1 (14.3%)	0 (0%)	7 (25.9%)
Histological subtype	pancreatobiliary	1 (100%)	0 (0%)	0 (0%)	1 (3.7%)
	intestinal	4 (50%)	3 (37.5%)	1 (12.5%)	8 (29.6%)
	gastric	15 (83.3%)	2 (11.1%)	1 (5.6%)	18 (66.7%)
Total		20 (74.1%)	5 (18.5%)	2 (7.4%)	

**Table 3:** CEC positivity by macroscopic, histological, and genetic IMPN characteristics.

		CEC (n, %)			
		Negative (n = 4)	Suspicious (n = 13)	Positive (n = 10)	*p*
IPMN type	branch-duct	0 (0%)	2 (33.3%)	4 (66.7%)	0.38
	mixed-type	2 (14.3%)	7 (50%)	5 (35.7%)	
	main-duct	2 (28.6%)	4 (57.1%)	1 (14.3%)	
Maximal dysplasia grade	low	3 (20%)	10 (50%)	7 (20%)	0.52
	intermediate	1 (20%)	3 (60%)	1 (20%)	
	high	0 (0%)	0 (0%)	2 (100%)	
Enhancing mural nodule	no	4 (16%)	12 (48%)	9 (36%)	0.78
	<5 mm	0 (0%)	1 (100%)	0 (0%)	
	≥5 mm	0 (0%)	0 (0%)	1 (100%)	
Thickened/enhancing cyst walls	no	4 (16.7%)	12 (50%)	8 (33.3%)	0.73
	yes	0 (0%)	1 (33.3%)	2 (66.7%)	
IPMN-induced acute pancreatitis	no	3 (15.8%)	10 (52.6%)	6 (31.6%)	0.85
	yes	1 (12.5%)	3 (37.5%)	4 (50%)	
Pancreatic head cyst with obstructive jaundice	no	4 (16%)	11 (44%)	10 (40%)	0.63
	yes	0 (0%)	2 (100%)	0 (0%)	
Increased serum level of CA 19-9	no	3 (15.8%)	9 (47.4%)	7 (36.8%)	0.74
	yes	0 (0%)	1 (33.3%)	2 (66.7%)	
Cyst growth-rate ≥5 mm/2 years (n = 13)	no	2 (25%)	3 (37.5%)	3 (37.5%)	1.0
	yes	1 (20%)	2 (40%)	2 (40%)	
Max. cyst size (mm)		18.8 (SD: 13.7)	15.8 (SD: 6.4)	17.7 (SD: 9.3)	0.79
KRAS genotype (CEC)	wild-type	4 (25%)	8 (50%)	4 (25%)	0.15
	mutated	0 (0%)	1 (20%)	4 (80%)	
KRAS genotype (tissue)	wild-type	3 (37.5%)	4 (50%)	1 (12.5%)	0.06
	mutated	1 (5.3%)	9 (47.4%)	9 (47.4%)	
Control patients (n = 5)		5	0	0	

**Table 4:** IF (PDX1, vimentin, EpCAM, L1CAM) by histological, and genetic IMPN characteristics.

		PDX1 (IF)		Vimentin		EpCAM		L1CAM	
		Positive	*p*	Positive	*p*	Positive	*p*	Positive	*p*
Histological subtype	pancreatobiliary	0 (0%)	0.23	0 (0%)	1.00	0 (0%)	0.55	0 (0%)	0.26
	gastric	7 (50.0%)		6 (42.9%)		10 (71.4%)		10 (71.4%)	
	intestinal	5 (83.3%)		2 (33.3%)		5 (71.4%)		6 (85.7%)	
CEC	suspicious	5 (41.7%)	0.18	1 (8.3%)	0.002	7 (58.3%)	0.38	8 (66.7%)	0.65
	positive	7 (77.8%)		7 (77.8%)		8 (80.0%)		8 (80.0%)	
Cluster	no	2 (50.0%)	0.17	2 (50.0%)	0.17	3 (60.0%)	0.44	3 (60.0%)	0.44
	yes	5 (100%)		5 (100%)		5 (100%)		5 (100%)	
KRAS (tissue)	wild-type	3 (75.0%)	0.60	0 (0%)	0.13	2 (50.0%)	0.57	2 (50.0%)	0.29
	mutated	9 (52.9%)		8 (47.1%)		13 (72.2%)		14 (77.8%)	

**Table 5:** KRAS mutation in CECs by macroscopic and histological IMPN characteristics.

		KRAS in CECs		
		Wild-Type	Mutation	*p*
IPMN type	branch-duct	2 (50%)	2 (50%)	0.21
	mixed-type	10 (90.9%)	1 (9.1%)	
	main-duct	4 (66.7%)	2 (33.3%)	
Max. cyst size (mm)		15.2 (SD: 8)	19.2 (SD: 10.5)	0.37
Histological subtype	pancreatobiliary	0 (0%)	1 (100%)	0.44
	gastric	11 (78.6%)	3 (21.4%)	
	intestinal	5 (83.3%)	1 (16.7%)	
Maximal dysplasia grade	low	13 (72.2%)	5 (27.8%)	0.55
	intermediate	3 (100%)	0 (0%)	
Enhancing mural nodule	no	15 (78.9%)	4 (21.1%)	0.43
	<5 mm	0 (0%)	1 (100%)	
	≥5 mm	1 (100%)	0 (0%)	
Thickened/enhancing cyst walls	no	14 (77.8%)	4 (22.2%)	1.0
	yes	2 (66.7%)	1 (33.3%)	
IPMN-induced acute pancreatitis	no	11 (73.3%)	4 (26.7%)	1.0
	yes	5 (83.3%)	1 (16.7%)	
Pancreatic head cyst with obstructive jaundice	no	15 (78.9%)	4 (21.1%)	0.43
	yes	1 (50%)	1 (50%)	
Increased serum level of CA 19-9	no	12 (85.7%)	2 (14.3%)	0.47
	yes	2 (66.7%)	1 (33.3%)	
Cyst growth rate ≥5 mm/2 years (n = 10)	no	5 (71.4%)	2 (28.6%)	1.0
	yes	2 (66.7%)	1 (33.3%)	

Max. = maximum; mm = millimeter; SD = standard deviation.

## Data Availability

Not applicable.

## Supplementary Materials

The following supporting information can be downloaded at: https://www.mdpi.com/article/10.3390/life13071570/s1, Figure S1: Positive and negative control for IHC Stain: Anti-EpCAM; Figure S2: Positive and negative control for IHC Stain: Anti-L1CAM; Figure S3: Positive and negative control for IHC Stain: Anti-PDX1; Figure S4: Anti-PDX1/-Vimentin-dual stain—Control stain with in vitro cultivated cell-lines; Figure S5: Anti-EpCAM/-L1CAM-dual stain—Control stain with in vitro cultivated cell-lines; Figure S6: Anti-PDX1/-Vimentin-dual stain—Control stain on white blood cells; Figure S7: Anti-EpCAM/-L1CAM-dual stain—Control stain on white blood cells.
