# Supplementary material for: Circulating Epithelial Cells in Patients with Intraductal Papillary Mucinous Neoplasm of the Pancreas
Source: Life (Basel). 2023 Jul 15;13(7):1570. doi: 10.3390/life13071570 (PMC10381561; doi:10.3390/life13071570)
Supplement: Supplementary file 1 [file life-13-01570-s001.zip › life-2394020-supplementary.pdf]

Figure S1: Positive and negative control for IHC Stain: Anti-EpCAM

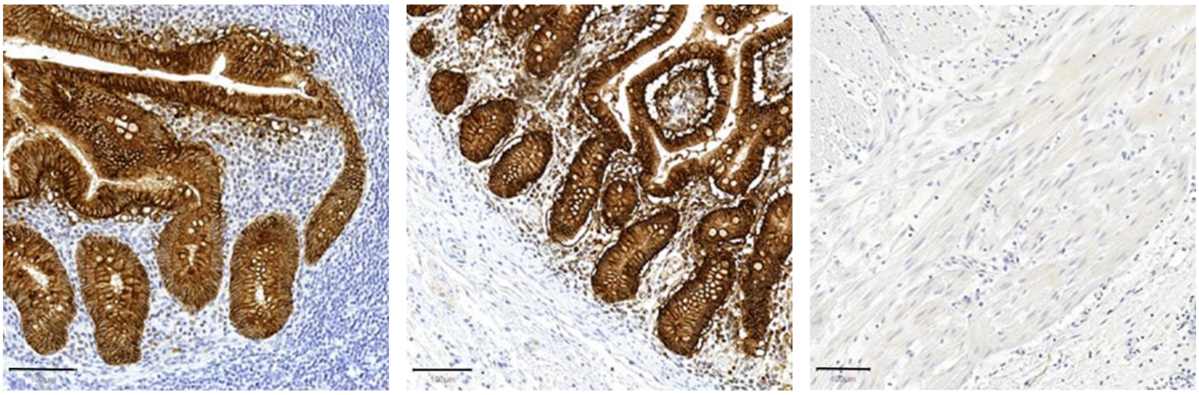

Appendix vermiformis [a] | Duodenum [a] | Smooth muscle [b]

Exemplary pictures of IHC-stain against EpCAM on tissues from Appendix, duodenum and smooth muscle, scale bar 100µm:

[a] Positive control: strong expression of EpCAM in the epithelia of the appendix and duodenum

[b] Negative control: no expression of EpCAM in smooth muscle as a tissue of non-epithelial origin.

Figure S2 Positive and negative control for IHC Stain: Anti-L1CAM

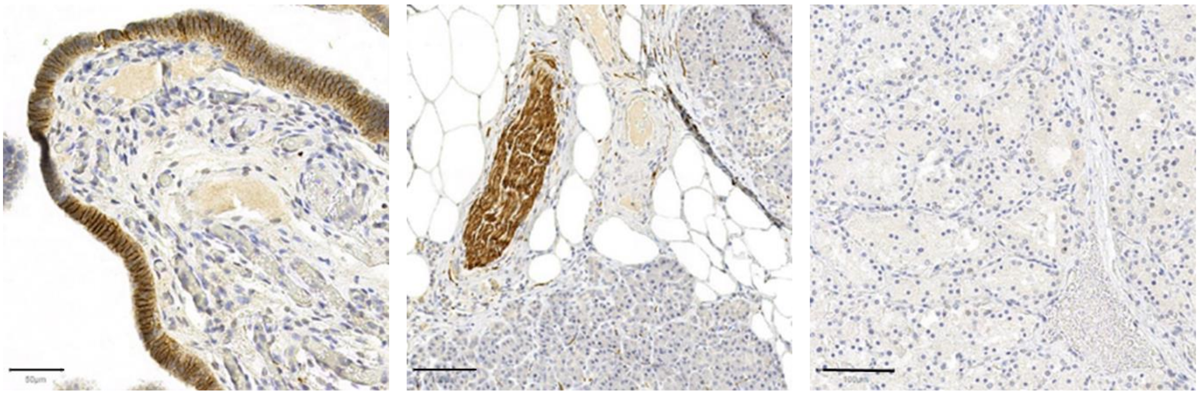

Fallopian tube[a] | Pancreas [b] | Thyroid gland [c]

Exemplary pictures of IHC-stain against L1CAM on tissues from fallopian tube, pancreas and thyroid gland:

[a] Positive control: strong expression of L1CAM in the epithelia of the fallopian tube; scale bar 50µm

[b] Positive control: nerve endings in pancreatic tissue show positive expression of L1CAM; scale bar 100µm

[c] Negative control: no expression of L1CAM in thyroid gland tissue, scale bar 100µm

Figure S3 Positive and negative control for IHC Stain: Anti-PDX1

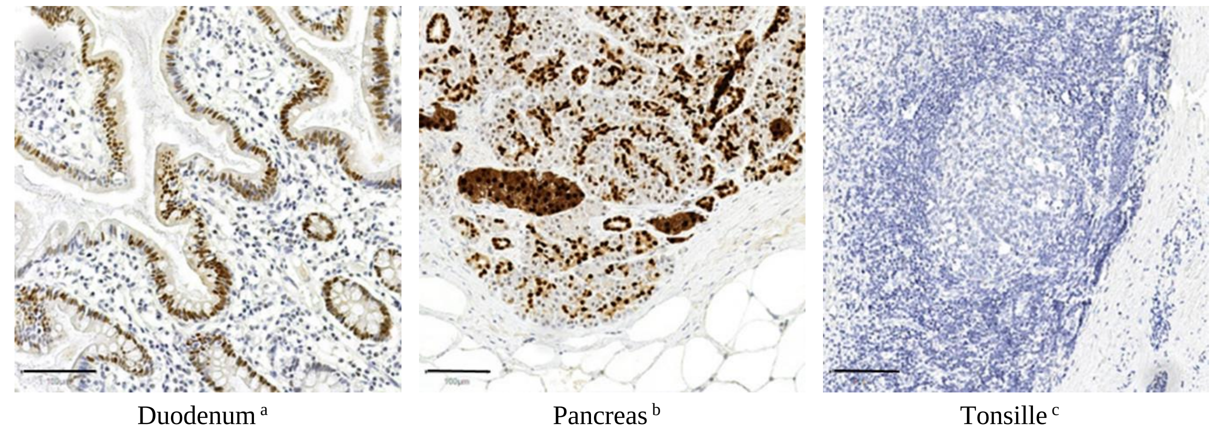

Exemplary pictures of IHC-stain against PDX1 on tissues from duodenum, pancreas and tonsille¸ scale bar 100µm:

[a] Positive control: epithelial cells in duodenum show moderate expression of PDX1.

[b] Positive control: strong expression of PDX1 in epithelial ductal cells and islets of Langerhans.

[c] Negative control: no PDX expression in tonsille tissue.

Figure S4: Anti-PDX1/-Vimentin-dual stain – Control stain with in vitro cultivated cell-lines

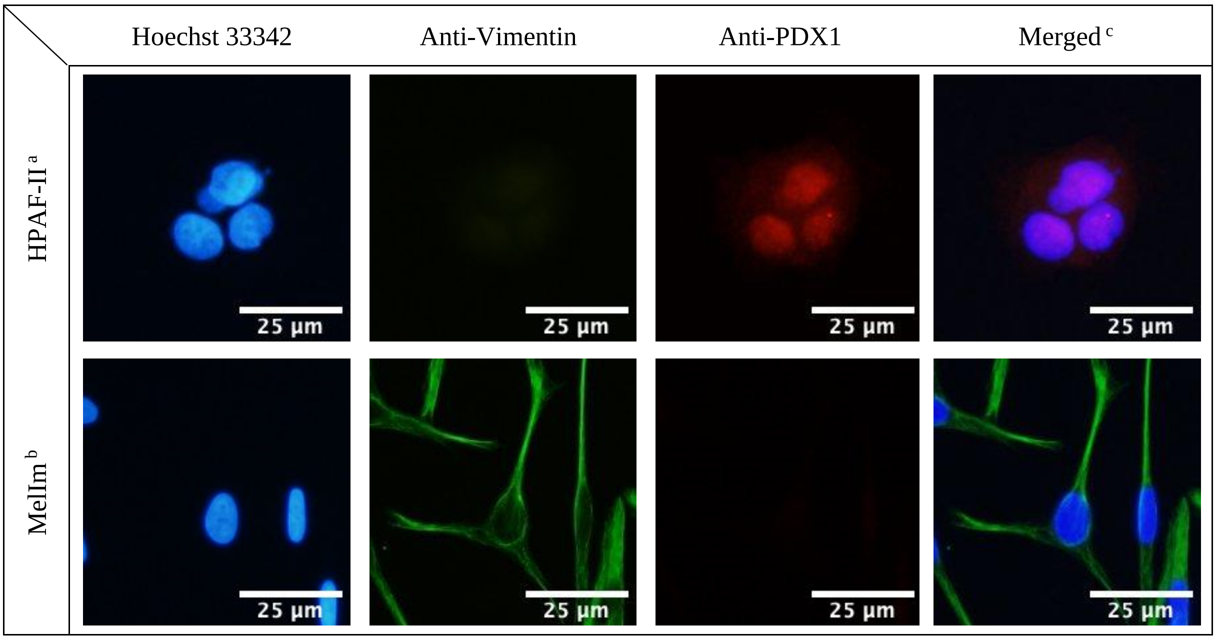

Secondary antibodies: Alexa Fluor® 488 and Cyanine3.

[a] HPAF-II-cells (pancreatic adenocarcinoma cell-line) show positive expression of PDX1 and no expression of Vimentin (negative control).

[b] MelIm-cells (melanoma-cell line) show positive expression of Vimentin and no expression of PDX1 (negative control)..

Figure S5: Anti-EpCAM/-L1CAM- dual stain - Control stain with in vitro cultivated cell-lines

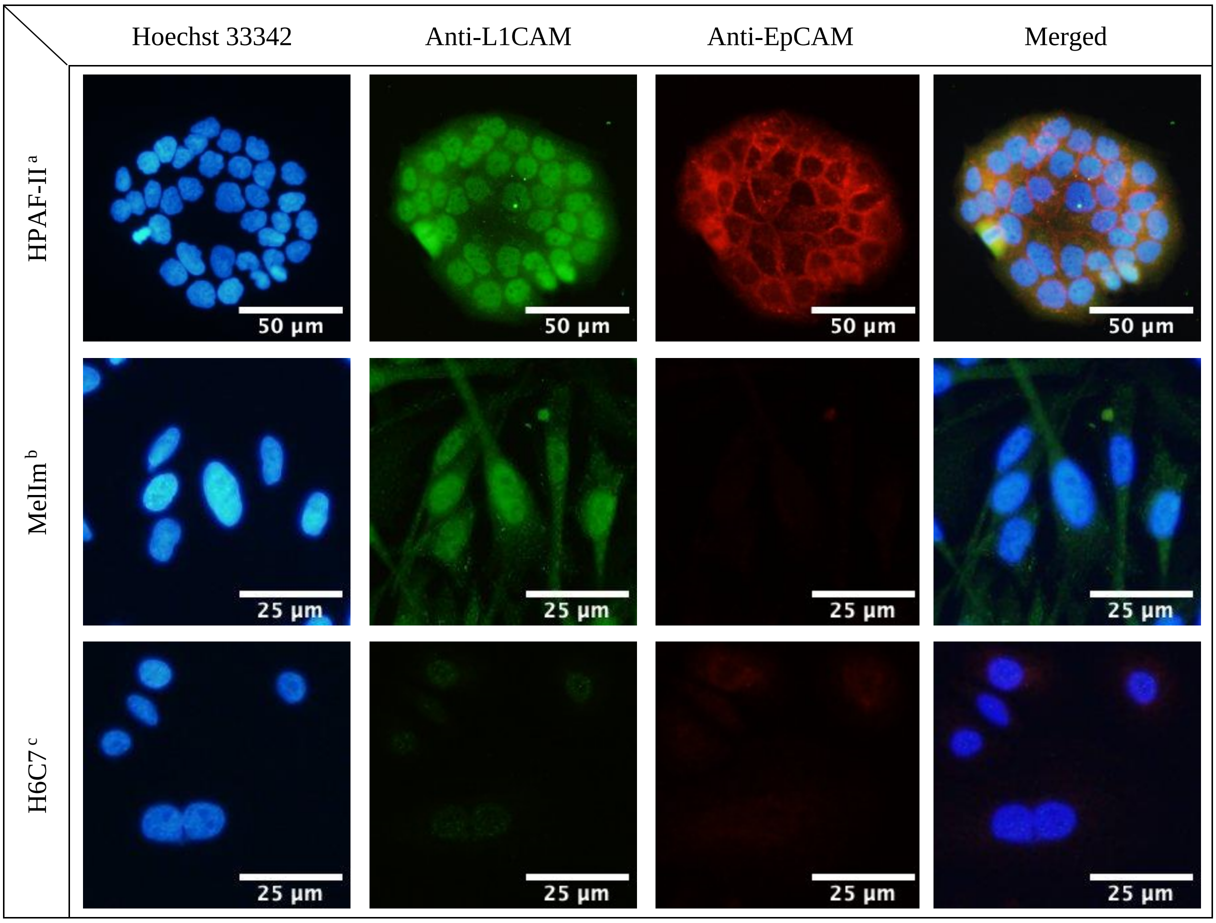

Secondary antibodies: Alexa Fluor® 488 and Cyanine3.

a HPAF-II-cells (pancreatic adenocarcinoma cell-line) show a positive stain for EpCAM and L1CAM

[b] MelIm-cells (melanoma-cell line) show a positive stain for L1CAM and no expression of EpCAM (negative control)

[c] H6C7-cells (human pancreatic duct epithelial cell line) show a very low expression of EpCAM and no expression of L1CAM (negative control).

Figure S6: Anti-PDX1/-Vimentin-dual stain – Control stain on white blood cells

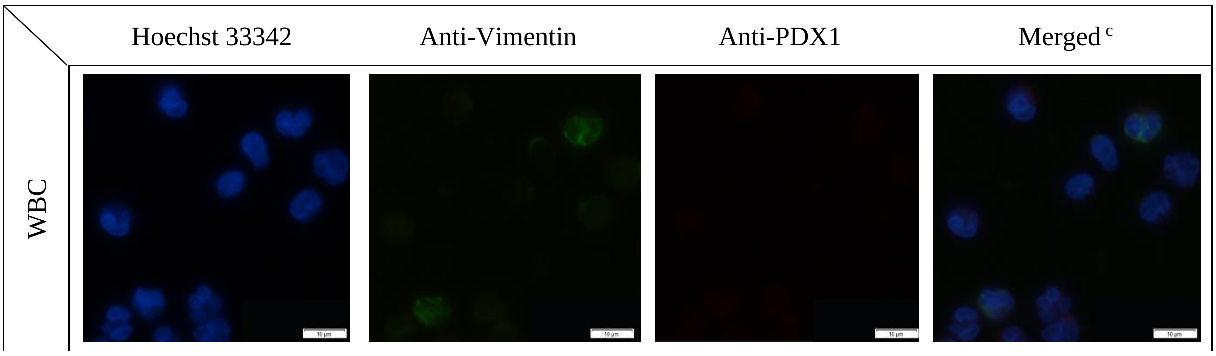

Exemplary pictures of the immunofluorescence labelling with PDX1 & Vimentin on white blood cells, from control patient. Secondary antibodies: Alexa Fluor® 488 and Cyanine3. 100× magnified; scale bar 10 µm; WBC= white blood cells. The WBCs show no expression of PDX1. Two WBCs show a very low vimentin expression.

Figure S7: Anti-EpCAM/-L1CAM- dual stain – Control stain on white blood cells

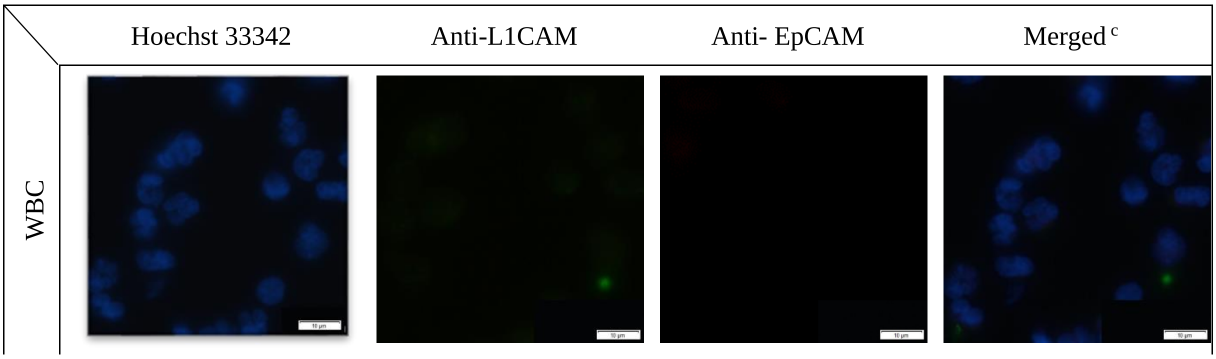

Exemplary pictures of the immunofluorescence labelling with Anti-EpCAM/-L1CAM on white blood cells, from control patient. Secondary antibodies: Alexa Fluor® 488 and Cyanine3. 100× magnified; scale bar 10 µm; WBC= white blood cells. The WBCs show no Anti-EpCAM and L1CAM expression.
